# An angiogenesis-related three-long non-coding ribonucleic acid signature predicts the immune landscape and prognosis in hepatocellular carcinoma

**DOI:** 10.1016/j.heliyon.2023.e13989

**Published:** 2023-02-23

**Authors:** Wenjuan Wang, Yingquan Ye, Xuede Zhang, Weijie Sun, Lingling Bao

**Affiliations:** a Department of Hematology and Oncology, Beilun District People's Hospital, Ningbo, China; b The First Affiliated Hospital of Anhui Medical University, Hefei, China; c Department of Oncology, Weifang People's Hospital, Weifang, China

**Keywords:** Hepatocellular carcinoma, Angiogenesis, Long noncoding RNA, Tumour immune microenvironment, Prognostic signature

## Abstract

The tumour microenvironment is a key determinant of the efficacy of immunotherapy. Angiogenesis is closely linked to tumour immunity. We aimed to screen long non-coding ribonucleic acids (lncRNAs) associated with angiogenesis to predict the prognosis of individuals with hepatocellular carcinoma (HCC) and characterise the tumour immune microenvironment (TIME). Patient data, including transcriptome and clinicopathological parameters, were retrieved from The Cancer Genome Atlas database. Moreover, co-expression algorithm was utilized to obtain angiogenesis-related lncRNAs. Additionally, survival-related lncRNAs were identified using Cox regression and the least absolute shrinkage and selection operator algorithm, which aided in constructing an angiogenesis-related lncRNA signature (ARLs). The ARLs was validated using Kaplan-Meier method, time-dependent receiver operating characteristic analyses, and Cox regression. Additionally, an independent external HCC dataset was used for further validation. Then, gene set enrichment analysis, immune landscape, and drug sensitivity analyses were implemented to explore the role of the ARLs. Finally, cluster analysis divided the entire HCC dataset into two clusters to distinguish different subtypes of TIME. This study provides insight into the involvement of angiogenesis-associated lncRNAs in predicting the TIME characteristics and prognosis for individuals with HCC. Furthermore, the developed ARLs and clusters can predict the prognosis and TIME characteristics in HCC, thereby aiding in selecting the appropriate therapeutic strategies involving immune checkpoint inhibitors and targeted drugs.

## 1 Introduction

Hepatocellular carcinoma (HCC) is the most widespread form of primary liver cancer and remains a worldwide burden [1]. Although the diagnostic and treatment modalities for HCC have been steadily evolving over the years, the prognosis for patients remains dismal, with an approximate 5-year survival rate of 18% [2]. Although current systemic regimens including targeted therapies and immunotherapy have great potential, the high heterogeneity of HCC reduces the effectiveness of systemic therapy and affects the precision of prognostic prediction [3]. Therefore, effective and specific biomarkers need to be identified for determining prognosis and providing personalised treatment in HCC.

Studies report that the tumour microenvironment (TME) influences tumour invasion and tumour response to immunotherapy [4]. Tumour neoangiogenesis is an essential marker of the TME and is extensively involved in the malignant biological behaviours of cancer [5]. Tumour cells promote angiogenesis and inflammation to evade monitoring by the body's immunity [6]. Therefore, exploring the correlation between tumour angiogenesis and the TME can help characterise distinct tumour immune profiles [7].

As biomarkers of cancer prognosis, long noncoding ribonucleic acids (lncRNAs) can be monitored dynamically during disease progression to better understand tumour biology [8]. Moreover, lncRNAs regulate mRNA expression during epigenetic processes and transcriptional stages. Additionally, they also participate in regulating tumour angiogenesis and mediating tumour inflammation and immune escape [[9], [10], [11], [12]]. Recent studies report that lncRNA PAARH promotes HCC progression and angiogenesis by activating the HIF-1α/VEGF signalling pathways [13]. Similarly, lncRNA SNHG22 also promotes HCC tumour angiogenesis and evolution via miR-16-5p methylation [14]. lncRNA MYLK-AS1 was also observed to facilitate HCC angiogenesis by targeting the miR-424-5p/E2F7 axis and triggering the VEGFR-2 pathway [9].

Recently, several studies have constructed signatures for the prediction of clinical outcomes in HCC [[15], [16], [17], [18]]. Notably, the angiogenesis-related lncRNA scoring system constructed by Lei et al. could better predict the prognosis of patients but could not characterise patients with different tumour immune microenvironments (TIME) [19]. Here, we construct an angiogenesis-related lncRNA signature (ARLs) to estimate TIME characteristics and prognosis of HCC, thereby distinguishing between ‘immune hot tumours’ and ‘immune cold tumours’. Furthermore, we aim to aid in the identification of immune checkpoint inhibitors (ICIs) and targeted therapies for individuals with HCC.

## 2 Materials and methods

### 2.1 Data collection

The transcriptome and relevant clinical data of individuals with HCC were retrieved from the TCGA-LIHC cohort of The Cancer Genome Atlas (TCGA) (https://portal.gdc.cancer.gov/). The Strawberry Perl script was applied to obtain the lncRNA and mRNA expression matrices from the downloaded transcriptome data. The transcriptome data of the CHCC cohort were obtained from the National Omics Data Encyclopedia (NODE) database (https://www.biosino.org/node) and the relevant clinical information was accessed from a previous study [20]. The set of angiogenesis-associated genes (AAGs) included in this study was derived from the marker gene set of the Molecular Signatures Database (https://www.gsea-msigdb.org/gsea/msigdb/index.jsp) (Table S1).

### 2.2 Identification of angiogenesis-related lncRNAs

The mRNA expression matrices of 36 AAGs were extracted using R software 4.1.2. Expression datasets of angiogenesis-associated lncRNAs were retrieved by means of co-expression algorithm based on Pearson correlation coefficient >0.4 and *p* < 0.001. The R was utilized to generate co-expression data for AAGs and angiogenesis-related lncRNAs, and the igraph package was applied to plot co-expression networks. Subsequently, patient survival and lncRNA expression data were combined for further analysis. The differentially expressed lncRNAs between tumour and normal tissues in patients with HCC were then extracted with the aid of the limma package (|Log2 fold change| > 1, *p* < 0.05). Furthermore, the volcano and heatmap of differential lncRNAs were plotted.

### 2.3 Establishment of an ARLs

The survival, survminer, glmnet, caret and timeROC packages were utilized to extract survival-associated lncRNAs in HCC to construct an angiogenesis-related signature. Survival-associated lncRNAs were extracted utilizing the univariate Cox regression (*p* < 0.01). The packages limma and corrplot were used to graph the link between the expression of survival-related lncRNAs, and plot an expression heatmap, respectively. Sankey plots were drawn using the ggalluvial and ggplot2 to analyse the regulatory role of prognosis-related lncRNAs on AAGs. All individuals were then randomly assigned into the training and testing groups (1:1 ratio). Subsequently, prognosis-related lncRNAs were extracted from the training group utilizing univariate cox regression algorithm. The least absolute shrinkage and selection operator (LASSO) was conducted to screen prognosis-related lncRNAs to avoid overfitting. Risk scores were calculated according to the coefficient (Coef) and expression (Expr) of each signature lncRNA using the following equation: $Risk\;score=\sum_{i=1}^{n}Coef\left(i\right)\times Expr\left(i\right)$. Finally, all patients were risk classified according to the median risk scores.

### 2.4 Stratification and validation of the ARLs

Cox regression analysis was utilized for the independent prognostic analysis of model risk scores and clinicopathological features in the TCGA and CHCC cohorts. Time-dependent receiver operating characteristic (ROC) curves of the training, testing, TCGA and CHCC cohorts was conducted to assess the value of the constructed ARLs. Kaplan-Meier (K-M) curves were plotted using the survivor and survminer packages for survival analysis in the different risk populations.

To stratify and validate the ARLs, individuals were classified into two groups based on stage, grade, gender, and age. The K-M curves of different clinical subgroups were then plotted to assess the suitability of the ARLs for individuals with different clinical subgroups. Additionally, the ggpubr and limma were used to analyse the risk scores of patients according to their clinicopathological parameters (age, grade, stage, and gender) and visualise the results, respectively. The ComplexHeatmap package was used to draw state heatmaps of risk groups and clinicopathological parameters.

### 2.5 Nomogram construction

Based on the Cox regression analysis, tumor stage and risk scores were used to construct a nomogram. Additionally, calibration curves for the Hosmer-Lemeshow test were plotted to evaluate the predicted and actual outcomes (method = ‘boot’, B = 1000). The survival, regplot, and rms packages were applied in this process.

### 2.6 Gene set enrichment analysis (GSEA)

The GSEA is an algorithm that uses the current information on the location, biological function of genes [21]. To examine the enrichment of pathways in different risk groups, GSEA was performed using the GSEA software. The grid, ggplot2 and gridExtra packages were employed to plot enrichment graphs.

### 2.7 Correlation between the ARLs and TIME

To explore the correlation between the ARLs and TIME of individuals with HCC, the ggtext, scales, ggpubr, tidyverse and ggplot2 packages were used to plot a bubble map of the links between model risk scores and immune cells. Additionally, the relative abundance of immune cells in the TIME was quantified by means of single sample GSEA (ssGSEA) [22]. The packages GSVA and GSEABase were applied to derive immune cell scores and immune function scores for every patient. Additionally, box plots were drawn to visualise the results using the packages reshape2 and ggpubr.

Immune checkpoint therapy is a novel weapon in cancer treatment, wherein it strengthens the anti-tumour immune response [23]. However, only a subset of patients with a specific tumour type respond to immune checkpoint therapy. One of the main challenges to this treatment is the lack of reliable predictive biomarkers [24]. Thus, to assess the potential predictive value of the ARLs in determining the efficacy of ICIs, we used the ggpubr and ggplot2 packages to analyse the expression of different marker genes in the risk groups and draw box plots. The binding of immune checkpoint programmed cell death-1 (PD-1) to programmed death-ligand 1 (PD-L1) contributes to cancer immune escape and is the most classical immune checkpoint pathway [25]. In some cases, there is a correlation between the expression of PD-L1 on tumour cells and clinical response to immunotherapy [26,27]. Cytotoxic T-lymphocyte-associated antigen 4 (CTLA-4) is also a classic immune checkpoint that negatively regulates immune function during the activation of immune effector cells [28]. Using the limma package, the three classical immune checkpoints expressed in both risk groups were investigated. The outcomes were observed using ggpubr package.

### 2.8 Drug sensitivity analysis

pRRophetic is a tool that predicts the chemotherapeutic response of individuals at the tumour gene level. Herein, a ridge regression model was applied using genes as predictors and drug sensitivity scores as outcome variable [29]. Furthermore, the risk models and the clinical implications of different HCC subtypes for drug therapy were explored. The pRRophetic and ggpubr packages were employed to obtain the half-maximum inhibitory concentration (IC50) of chemicals and targeted agents in the different risk populations and draw a box plot for the drugs showing a significant difference (*p* < 0.001), respectively.

### 2.9 Consensus clustering analysis

The ConsensusClusterPlus package was utilized for consensus clustering analysis of the patients in the TCGA-LIHC cohort according to the developed signature. Principal component analysis (PCA) was carried out using the ggplot2 and Rtsne packages, and the associations between different HCC subtypes with patient survival and the TIME were further explored. The ESTIMATE tool evaluates the number of stromal and immune cells infiltration in tissues [30]. In this study, the ESTIMATE package was utilized to determine the relative quantities of stromal and immune cells in each patient's tumour tissue to obtain the stromal and immune scores. The two scores were summed to obtain the ESTIMATE scores, which negatively correlate with tumour purity. Finally, box plots of the ESTIMATE analysis for different HCC clusters were plotted using the ggpubr package.

## 3 Results

### 3.1 Angiogenesis-related lncRNAs in HCC

**Fig. 1:**
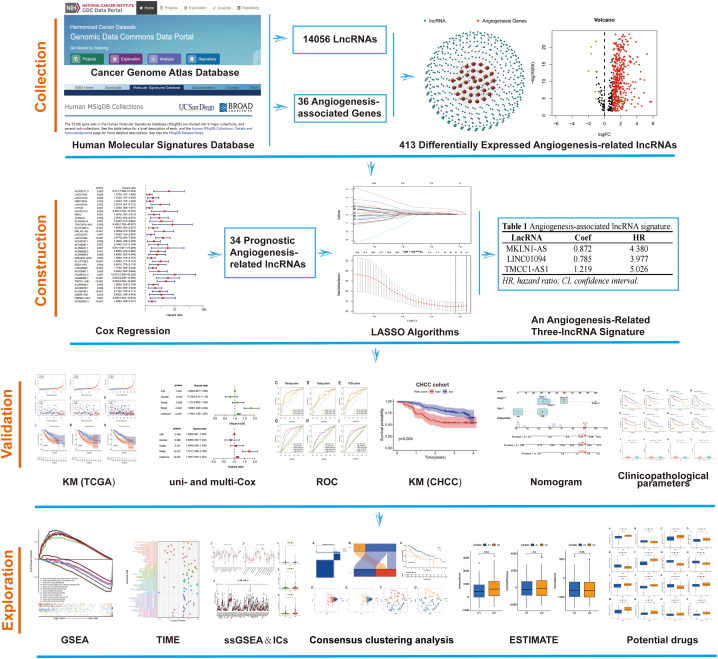
Flow diagram of the study scheme. 14056 lncRNAs and 36 AAGs were obtained from TCGA and MsigDB. Afterward, 575 angiogenesis-related lncRNAs were found, of which 413 were differentially expressed. Furthermore, a three-lncRNA signature was constructed using Cox regression and LASSO algorithm. The signature was then validated using K–M, ROC curves and Cox regression. Furthermore, the CHCC cohort was used to validate this signature. Finally, cluster analysis was applied to divide the dataset into two clusters according to distinct TIME subtypes.

**Fig. 2:**
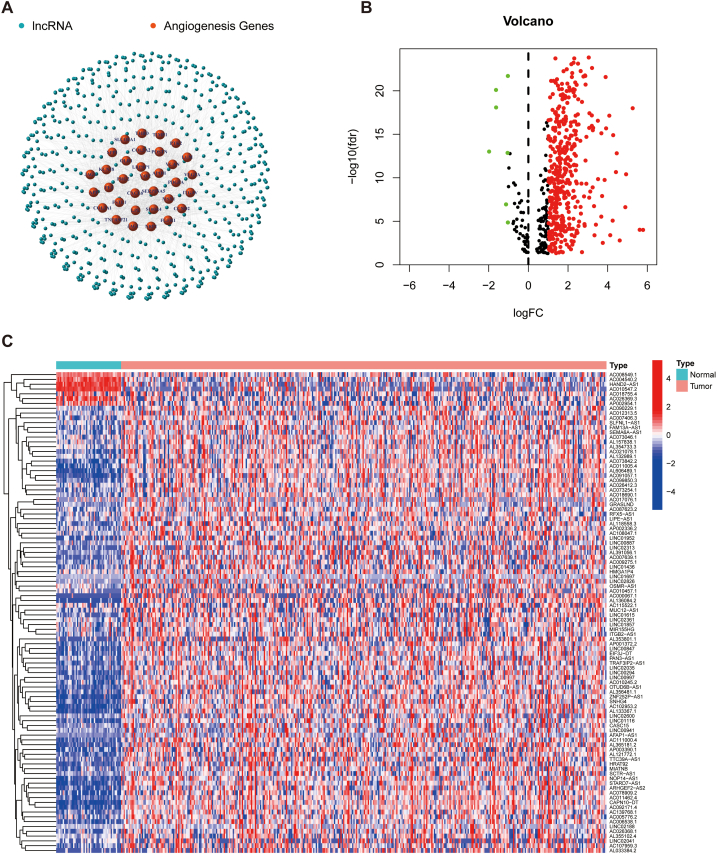
Angiogenesis-related lncRNAs in HCC. A Correlation network diagram of AAGs and angiogenesis-associated lncRNAs. B Volcano graph of 413 angiogenesis-related lncRNAs with differential expression. C Heat map of differentially expressed angiogenesis-related lncRNAs in tumor and normal tissues.

The study scheme is illustrated in Fig. 1 in the form of a flow diagram. The transcriptomic data were obtained from the TCGA-LIHC cohort. Based on co-expression algorithm, we obtained a total of 575 lncRNAs associated with the 36 AAGs, and constructed a co-expression network of angiogenesis-associated lncRNAs and AAGs (Fig. 2A). Furthermore, a total of 413 angiogenesis-related lncRNAs displayed differential expression between HCC tumours and normal samples. Notably, seven lncRNAs were down-regulated in tumour tissues (Fig. 2B). Additionally, the heatmap explains the expression of angiogenesis-related lncRNAs in HCC (Fig. 2C).

### 3.2 Signature construction and validation

**Fig. 3:**
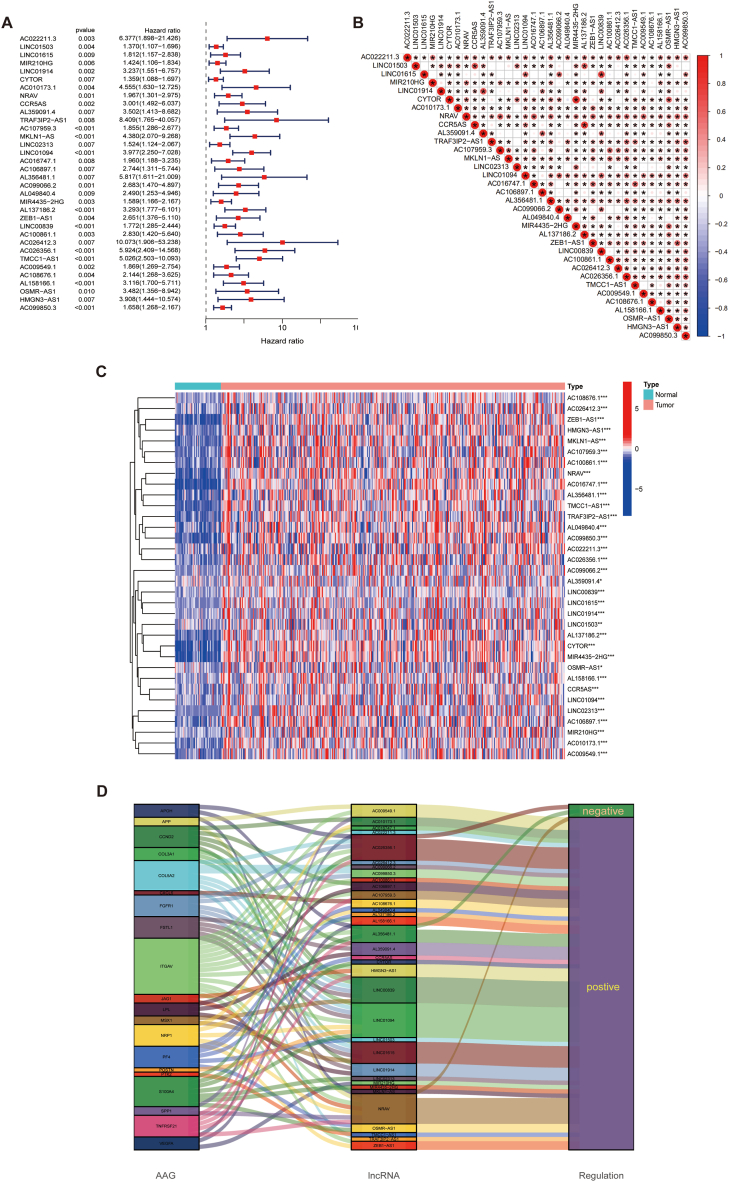
Angiogenesis-related lncRNAs associated with HCC prognosis. A The 34 angiogenesis-related lncRNAs extracted utilizing univariate Cox regression. B Expression correlation between the 34 angiogenesis-associated lncRNAs. C Heatmap of prognostic angiogenesis-associated lncRNAs. D The Sankey of angiogenesis-associated lncRNAs and angiogenesis-associated genes. *p < 0.05, **p < 0.01, and ***p < 0.001.

**Fig. 4:**
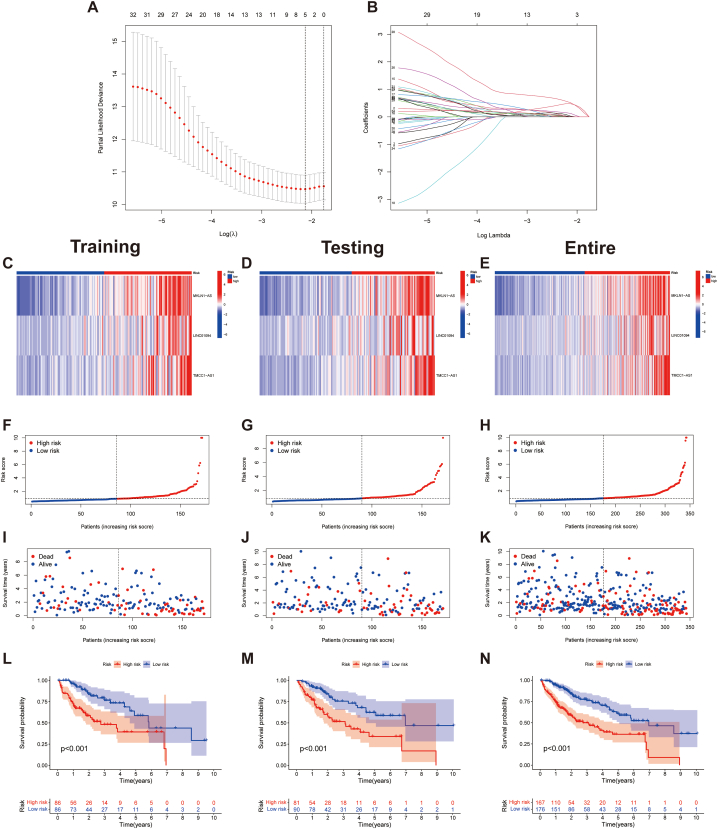
Assessment of the prognostic signature. A-B The coefficient and partial likelihood deviance of the ARLs. C-E Heat map of expression of the three lncRNAs in the training, testing, and entire cohorts. F–H Risk distribution in the three cohorts. I–K Survival time and status in the three cohorts. L-N K-M curves for OS in the three cohorts.

Cox analysis identified 34 angiogenesis-associated lncRNAs that were linked to survival (Fig. 3A). The expression of these lncRNAs in HCC is broadly correlated (Fig. 3B). Notably, all of these lncRNAs were upregulated in HCC tumor tissues (Fig. 3C). Furthermore, as presented in the Sankey plot, most of the lncRNAs were positively regulated for AAGs; however, AC026356.1, AL356481.1, and NRAV showed a partial negative regulation (Fig. 3D). LASSO regression analysis (Fig. 4A and B), further identified three angiogenesis-related lncRNAs that were used to construct the ARLs (Table 1).

**Table 1:** Angiogenesis-related lncRNA signature.

lncRNA	Coef	HR	HR (95%CI)	*p*-value
MKLN1-AS	0.872	4.380	2.070–9.268	<0.001
LINC01094	0.785	3.977	2.250–7.028	<0.001
TMCC1-AS1	1.219	5.026	2.503–10.093	<0.001

HR, hazard ratio; CI, confidence interval.

**Fig. 5:**
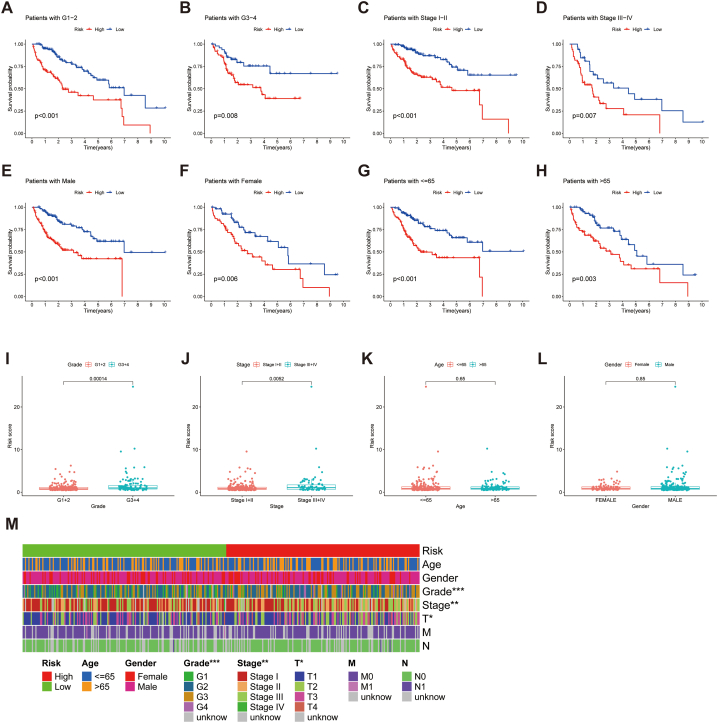
K-M curves of the high- and low-risk groups were stratified by A, B grade, C, D TNM stage, E, F sex, and G, H age. Patients with different levels of risk scores sorted by I grade, J stage, K age, and L gender. M A strip chart of the associations between clinicopathological features and risk groups. *p < 0.05, **p < 0.01, and ***p < 0.001.

Patients with HCC were classified into high- and low-risk groups according to the median value of the risk score. The heat map revealed that all three lncRNAs were expressed at high levels in the high-risk group (Fig. 4C–E). Furthermore, the TCGA cohort was sorted into training and testing groups, and clinicopathological features of individuals in both groups were compared (Table 2). We further assessed the survival status, survival time, and risk score distribution of patients in the training, testing, and entire cohorts in the risk groups. The individuals in the low-risk group displayed a better prognosis compared to those in the high-risk group across different cohorts (Fig. 4F-N). The stratification results revealed that low-risk groups (with different tumour grades, TNM stages, gender and age) had better survival than the high-risk groups (Fig. 5A–H); this demonstrated the stability and applicability of the model. A comparison of risk scores between different clinicopathological parameters showed that the scores for grades 3 and 4 and TNM stages III and IV were higher than those for grades 1 and 2 and TNM stages I and II, respectively; however, these risk scores did not differ significantly across age and gender (Fig. 5I-L). Moreover, the status heatmap of clinicopathological parameters showed that tumour grade, TNM stage and T stage differed across the low- and high-risk groups (Fig. 5M).

**Table 2:** Comparison of clinicopathological parameters between the testing and training cohorts.

Covariates	Type	Total	Testing cohort	Training cohort	p-value
Age	≤65	216 (62.97%)	114 (66.67%)	102 (59.3%)	0.1935
	>65	127 (37.03%)	57 (33.33%)	70 (40.7%)	
Gender	Female	110 (32.07%)	51 (29.82%)	59 (34.3%)	0.4397
	Male	233 (67.93%)	120 (70.18%)	113 (65.7%)	
Grade	G1	53 (15.45%)	21 (12.28%)	32 (18.6%)	0.4886
	G2	161 (46.94%)	83 (48.54%)	78 (45.35%)	
	G3	112 (32.65%)	57 (33.33%)	55 (31.98%)	
	G4	12 (3.5%)	6 (3.51%)	6 (3.49%)	
	unknow	5 (1.46%)	4 (2.34%)	1 (0.58%)	
Stage	Stage I	161 (46.94%)	86 (50.29%)	75 (43.6%)	0.5998
	Stage II	77 (22.45%)	39 (22.81%)	38 (22.09%)	
	Stage III	80 (23.32%)	36 (21.05%)	44 (25.58%)	
	Stage IV	3 (0.87%)	1 (0.58%)	2 (1.16%)	
	unknow	22 (6.41%)	9 (5.26%)	13 (7.56%)

### 3.3 Model evaluation

**Fig. 6:**
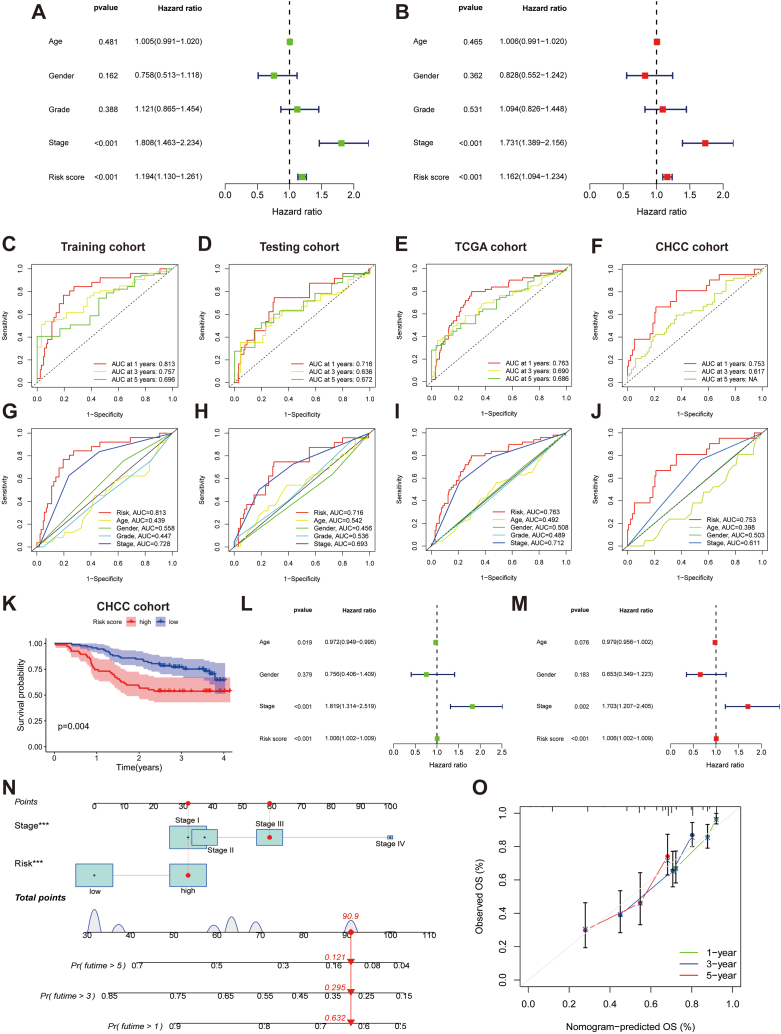
Validation of the ARLs in HCC. A Forest plots for univariate and B multivariate Cox regression in the TCGA cohort. C–F ROC curves of 1-, 3-, and 5-year survival for the ARLs in the training, testing, TCGA, and CHCC cohorts. G-J ROC curves for the ARLs and different clinicopathological parameters in the different cohorts. K K–M curve for overall survival in the CHCC cohort. L Forest plots for univariate and M multivariate analysis in the CHCC cohort. N Nomogram for predicting survival. O The calibration curves of the nomogram. *p < 0.05, **p < 0.01, and ***p < 0.001.

The risk score was established as a significant independent predictor of patient prognosis in the TCGA cohort as per both univariate and multivariate Cox analyses, with hazard ratio values of 1.194 (95% confidence interval 1.130–1.261) and 1.162 (95% confidence interval 1.094–1.234) respectively (*p* < 0.001) (Fig. 6A and B).

To further verify the robustness of the signature, ROC curves were utilized to assess the specificity and sensitivity of the signature in predicting the clinical outcomes of HCC individuals. The area under the curve values for the signature predicting overall survival (OS) at 1-, 3-, and 5- years in the training cohort were 0.813, 0.757, and 0.696, respectively (Fig. 6C); 0.716, 0.636, and 0.672 in the validation cohort (Fig. 5D); 0.763, 0.690 and 0.686 in the TCGA cohort, respectively (Fig. 6E). Furthermore, the area under the curve values of the ARLs for predicting OS at 1- and 3- years in the CHCC cohort were 0.753 and 0.617, respectively (Fig. 6F). Additionally, the area under the curve values for the tumour stage at 1 year for the training, testing, TCGA and CHCC cohorts were 0.728, 0.693, 0.712, and 0.611, respectively (Fig. 6G–J). K-M curves showed that low-risk patients in the CHCC cohort had a better prognosis (*p* = 0.004) (Fig. 6K). Notably, the risk score acted as a significant independent predictor of prognosis for the CHCC cohort (*p* < 0.001) (Fig. 6L and M).

### 3.4 Nomogram construction and validation

According to the multivariate Cox, the TNM stage and risk score demonstrated a significant impact on the clinical outcomes of HCC. We, therefore, constructed a nomogram according to these variables to help predict the prognosis of individuals (Fig. 6N). Corresponding scores for each factor (stage and risk score) were derived from the nomogram, wherein the total score served as a predictive tool for prognosis. We estimated the OS rates for a patient with high-risk and Stage III at 1-, 3- and 5-year to be 0.632, 0.295, and 0.121, respectively. The calibration curves demonstrated good consistency between the predicted results of the nomogram and actual situation (Fig. 6O).

### 3.5 GSEA

**Fig. 7:**
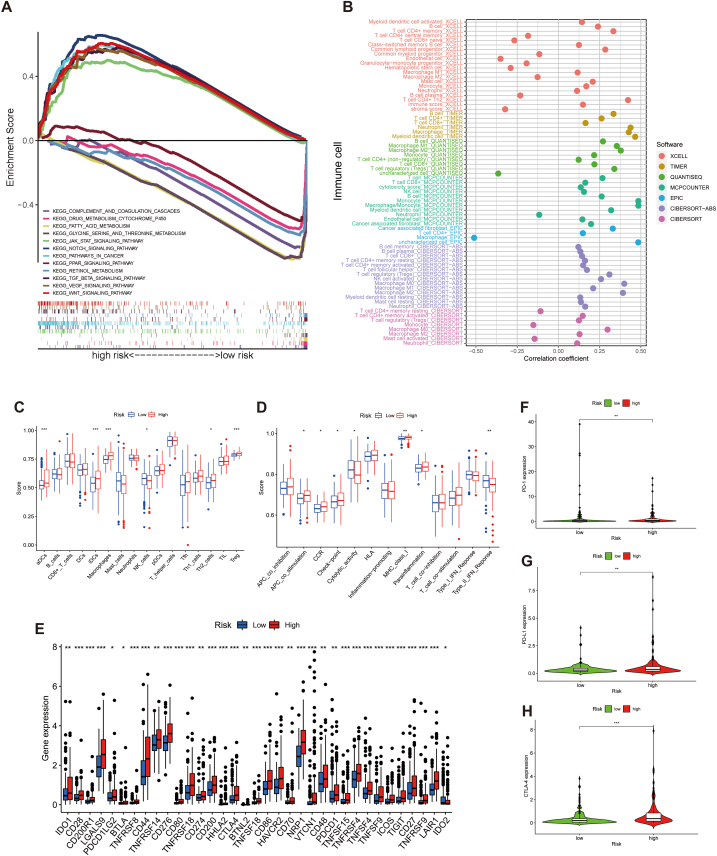
GSEA and immune correlation analysis. A The different pathways were enriched in both risk groups. B Bubble chart of the link between risk scores and immune cells. C Boxplots of immune cell enrichment scores in the two risk groups. D Boxplots of immune-related functional enrichment scores in the different risk groups. E Comparison of immune checkpoints between the two risk populations. F–H Expression of PD-1, PD-L1, and CTLA-4 in different risk populations. *p < 0.05, **p < 0.01, and ***p < 0.001.

GSEA revealed that the VEGF, TGF-BETA, WNT, JAK-STAT, and NOTCH pathways were enriched in the high-risk population (Fig. 7A); all of these pathways were involved in malignant invasion and tumor angiogenesis [[31], [32], [33], [34], [35]]. Contrastingly, complement and coagulation cascades, cytochrome P450-related metabolic functions, fatty acid, and retinol metabolic functions were enriched in the low-risk population.

### 3.6 Prognostic signature for predicting the TIME

To explore the underlying predictive power of the signature in regard to the TIME in HCC, we first investigated the link between immune cells and risk scores. As shown in the bubble chart (Fig. 7B), the majority of immune effector cells, including CD8^+^ T cells, were positively correlated with the risk scores; however, the outcomes for macrophages and neutrophils were inconsistent across platforms. Additionally, the risk scores correlated positively with immune scores and negatively with stroma scores on the XCELL platform.

ssGSEA analysis revealed a higher proportion of activated dendritic cells, immature dendritic B cells, macrophages, T helper type 2 cells, and regulatory T cells in the high-risk population. Contrarily, the NK cell population was lower in the high-risk population (Fig. 7C). As for immune effect, antigen-presenting cell co-stimulation, chemokine receptor function, immune checkpoints, major histocompatibility complex class I activity, and parainflammation were significantly weaker in the low-risk population; however, the opposite was true for cytolytic activity and type II interferon responses (Fig. 7D). For the immune checkpoints (Fig. 7E), mRNA expression of most immune checkpoint genes was significantly overexpressed in the high-risk population, indicating a highly immunosuppressed state in the high-risk population. The ICIs currently used in clinical practice mainly target CTLA-4, PD-1, and PD-L1 [24]. As shown in the violin plots (Fig. 7F–H), these three immune checkpoints were expressed at a considerably higher level in the high-risk group, suggesting a better efficacy of ICIs in patients with HCC who are predicted to be at high risk using the ARLs [36].

### 3.7 Prediction of drug sensitivity by the angiogenesis-associated lncRNA signature

**Fig. 8:**
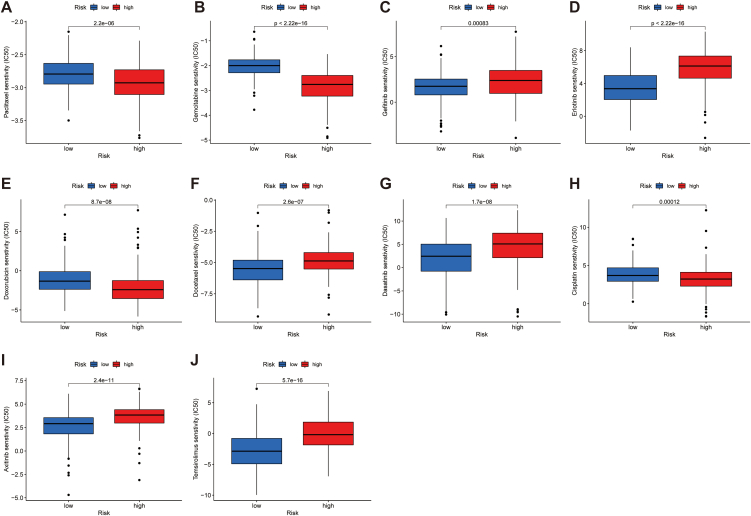
Drug sensitivity analysis. A-J Boxplots of IC50 values for chemotherapeutic and targeted agents in low- and high-risk populationa.

Drug sensitivity analysis revealed significantly higher IC50 values for the chemotherapeutic agents cisplatin, doxorubicin, paclitaxel and gemcitabine in the low-risk population. In contrast, docetaxel treatment yielded opposite results. With regard to targeted agents, the IC50 values of axitinib, erlotinib, dasatinib, gefitinib and temsirolimus were lower in the low-risk population (Fig. 8A–J).

### 3.8 Consensus clustering based on the ARLs

**Fig. 9:**
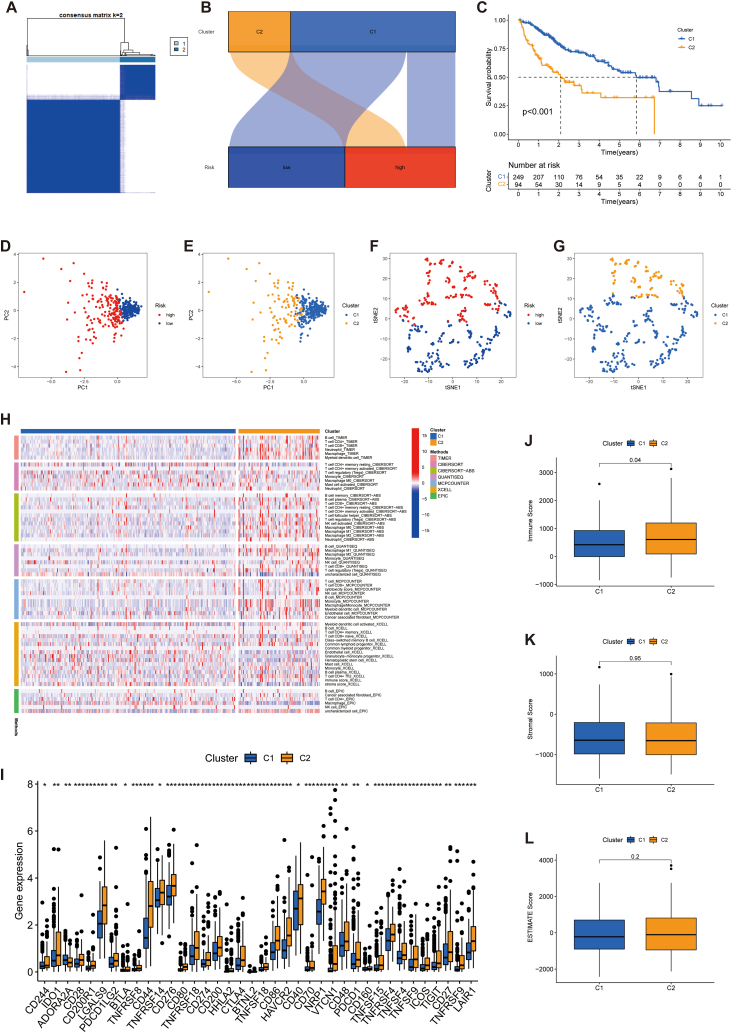
HCC classification based on the ARLs. A Individuals were divided into two clusters. B Sankey diagram of the association between the risk groups and two clusters. C K-M curves of the two clusters. D-G PCA and tSNE analyses of the clusters and risk populations. H Heat map of the amount of different types of immune cells. I Expression of immune checkpoint markers in the two clusters. J-L Stromal scores, immunity scores, and ESTIMATE scores in both clusters. *p < 0.05, **p < 0.01, and ***p < 0.001.

**Fig. 10:**
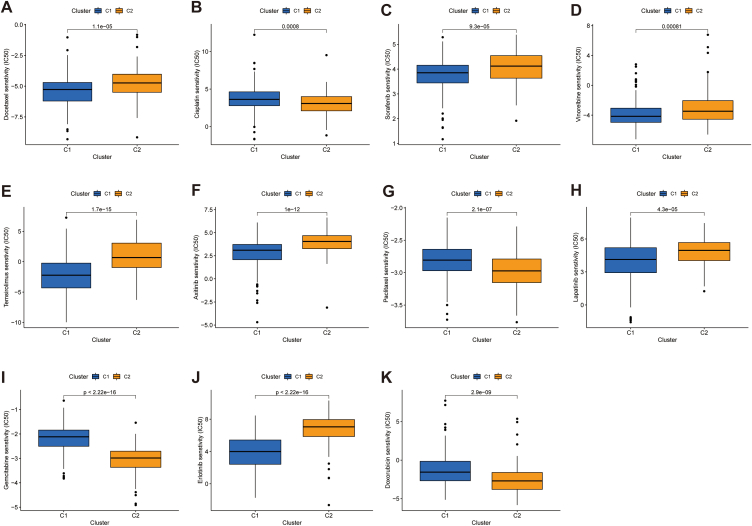
IC50 analysis based on the different clusters. A-K Boxplots of IC50 values for different drugs in the two clusters.

It has been demonstrated that tumour subgroups obtained using cluster analysis have different immune microenvironmental characteristics that influence the efficacy of tumour immunotherapy [37,38]. In this research, we used cluster analysis based on predictive signatures to divide the individuals in the TCGA-LIHC cohort into two clusters (with small correlations between the two clusters and large correlations within them) (Fig. 9A). The results showed that most of the individuals in cluster 1 belonged to the low-risk group, whereas all patients in cluster 2 were in the high-risk group (Fig. 9B). Survival analysis indicated that individuals in cluster 1 had a significantly better prognosis than those in cluster 2 (*p* < 0.001) (Fig. 9C). These findings indicate that the constructed cluster typing can predict the clinical outcomes of HCC individuals. Furthermore, PCA and tSNE analyses clearly distinguished the distributional characteristics of the two clusters (Fig. 9D–G). Additionally, the heat map revealed that most immune cells were more abundant in cluster 2 (Fig. 9H). Regarding immune checkpoints, the box plots suggested that most immune checkpoint genes expressed at a significantly higher level in cluster 2, indicating that tumours in cluster 2 were highly immunosuppressed. This could result in superior treatment response to ICIs in patients in cluster 2 than those in cluster 1 (Fig. 9I). Finally, ESTIMATE revealed that cluster 2 samples had higher immunity scores than that of cluster 1; however, no significant difference was observed between the clusters in terms of stromal scores (Fig. 9J-L). Additionally, there was a difference in IC50 values for multiple chemotherapeutic and targeted anti-tumour drugs between clusters 1 and 2 (Fig. 10A–K). Notably, the IC50 of sorafenib, a first-line drug for advanced HCC, was significantly higher in cluster 2, suggesting that our clustering analysis could aid in the selection of targeted therapeutic regimens for advanced HCC. Thus, these results suggest that HCC cluster typing based on the characteristics of angiogenesis-associated lncRNAs can not only determine the TIME but also aid in the clinical selection of ICIs, targeted drugs, and chemotherapeutic agents.

## 4 Discussion

The induction of angiogenesis is the most essential hallmark of cancer [39]. As tumor growth requires a supply of nutrients and oxygen, angiogenesis is continuously active to maintain tumour growth [40]. Angiogenic cytokines are a vital group of factors induce angiogenesis, performing an immune modulatory role [7]. Several angiogenic regulators and pathways have been reported to be dysregulated in HCC, playing a role in HCC progression [41]. Therefore, the survival of patients receiving sorafenib can be predicted by measuring angiogenic cytokines levels in the serum [42]. Angiogenic factors can also be involved in immunosuppression by suppressing immune effector cells and antigen-presenting cells [43]. Thus, tumour angiogenesis exerts an important influence in tumour immunity and progression.

The lncRNAs regulate the malignant hallmarks of HCC, such as resistance to cell death, uncontrolled proliferation, immune escape, metabolic reprogramming, and metastasis. Recent researches suggest that lncRNAs are involved in the induction of angiogenesis in HCC [9,44,45]. Although lncRNAs have been demonstrated to have high predictive power in predicting the clinical outcomes of patients with HCC [[46], [47], [48], [49], [50]], the relevance of angiogenesis-related lncRNAs in predicting the TIME of HCC remains unexplored.

In the present research, we constructed an ARLs to predict the clinical outcomes and TIME characteristics of individuals with HCC. Three angiogenesis-associated lncRNAs (MKLN1-AS, LINC01094, and TMCC1-AS1) were identified for the construction of angiogenesis-associated lncRNA signatures using Cox and LASSO regression analysis. Among these lncRNAs, MKLN1-AS were expressed at a high level in individuals with HCC and act as a sponge for miR-654-3p to promote HCC development and progression [51,52]. Moreover, TMCC1-AS1 expression has also been associated with OS and recurrence-free survival in HCC [53]; however, its regulatory mechanisms are yet to be elucidated. Previous research has indicated that LINC01094 can facilitate the progression of numerous cancers, including ovarian cancer, clear cell renal cell carcinoma, and glioma through various mechanisms [[54], [55], [56]]; however, its role in HCC remains unclear. Given the prognostic value of LINC01094 and TMCC1-AS1 in HCC, the molecular mechanisms of both deserve further validation. We further divided individuals with HCC into high- and low-risk groups. The results suggested that the risk scores of the ARLs can predict the OS independently and exhibit a good prognostic predictive effect. Additionally, the robustness of this signature is verified in the CHCC cohort. ROC curves revealed that the constructed lncRNA signature outperformed traditional clinicopathological parameters in predicting survival. Similarly, the constructed predictive nomogram showed good agreement between the observed and predicted rates of OS. Therefore, the developed ARLs can better predict the clinical outcomes of individuals with HCC.

As an important immunosuppressive signal in TIME, immune checkpoints can suppress the anti-tumour immune response, thereby allowing tumor cells to evade immune cell killing [57,58]. In the high-risk group, most immune checkpoint genes were expressed at a higher level compared to the low-risk population, suggesting strong immunosuppression in the former. This difference in expression levels could partially explain the better clinical outcomes of the low-risk population. Given that current ICIs primarily act on immune checkpoints, high-risk individuals may obtain more benefit from ICIs. Additionally, the lncRNA signature also provides a basis for the selection of targeted drugs and chemotherapeutics for HCC treatment. Among these, axitinib, a highly selective VEGF receptor inhibitor, has been reported to have anti-tumour activity in combination with the PD-L1 inhibitor avelumab in patients with advanced HCC [59,60]. The IC50 values suggest that axitinib might be more effective in individuals in the low-risk group. Erlotinib, a TKI that primarily targets the epidermal growth factor receptor; a meta-analysis showed that erlotinib in combination with the anti-angiogenesis-targeting agent, bevacizumab, is effective for the second-line therapy of advanced HCC [61]. Furthermore, the combination of gefitinib, an epidermal growth factor receptor inhibitor, and lenvatinib, an anti-tumour angiogenesis TKI, displayed potent anti-proliferative effects in epidermal growth factor receptor-expressing HCC cell lines in vitro. Additionally, the combination of the two agents also produced significant clinical responses in 12 patients with advanced HCC who were resistant to lenvatinib [62]. Consistently, this study also indicates that gefitinib and erlotinib might be more effective for the low-risk population.

Molecular subtypes of tumours are associated with patient clinical outcomes and the TIME [63]. To analyse differences in survival and TIME characteristics of individuals with varied subtypes of HCC, we classified patients into two clusters based on the constructed angiogenesis-associated lncRNA signature. Cluster 1 was mostly classified as a low-risk population, while all cases in cluster 2 were classified into the high-risk patients. The survival of individuals in cluster 1 was better than those in cluster 2. Most immune cells and immune checkpoints were abundant and highly expressed in cluster 2 than in cluster 1, forming an immunosuppressive TIME that could contribute to the immune evasion of tumour cells. In the majority of tumours, ‘cold tumours’ refer to tumours with low infiltration of effector immune cells, which contributes to the low efficiency of ICIs treatment [64]. Contrastingly, ‘hot tumours’ refer to tumours with high infiltration of immune effector cells, particularly the CD8^+^ T cells [65], which are more sensitive to ICIs [66]. These findings suggest that patients from cluster 2 can be considered to have ‘hot tumours’ and may more likely benefit from ICIs. In patients with advanced HCC, sorafenib remains the first-line treatment as an anti-angiogenic TKI. Drug sensitivity analysis suggested that patients from cluster 1 would be more sensitive to sorafenib. Additionally, the combination of temsirolimus, an allosteric mammalian target of rapamycin inhibitor, and doxorubicin, a chemotherapeutic agent, has been reported to enhance the anti-tumour effect in HCC cells [67]. In the present study, cluster 1 was more sensitive to temsirolimus, while showing contrary reactions to doxorubicin. Thus, our cluster analysis not only facilitates the prediction of clinical outcomes and TIME features of different subtypes of HCC but also provides a basis for determining personalised therapeutic strategies.

Although the ARLs was verified by various approaches, there are certain limitations of this study. First, in retrospective studies, bias could be present in the cases under study, which cannot be assessed. Second, the mechanism underlying angiogenesis-related lncRNA in the signature requires further experimental validation.

## 5 Conclusions

The ARLs and clusters established in this research can effectively predict the clinical outcomes and TIME characteristics of individuals with HCC. These findings provide a foundation for the selection of ICIs, TKIs, and chemotherapeutic agents in clinical settings. However, the role of the ARLs in predicting the efficacy of ICIs in HCC treatment requires further analysis using prospective clinical studies.

## Author contribution statement

Wenjuan Wang and Yingquan Ye: Performed the experiments; Analyzed and interpreted the data; Write the paper.

Xuede Zhang and Weijie Sun: Contributed the analysis tools and data.

Lingling Bao: Conceived and designed the experiments.

## Funding statement

This work was supported by the Natural Science Foundation of Ningbo City (2019A610235).

## Data availability statement

The data sets used and/or analyzed during the current study are available from the corresponding author upon reasonable request.

## Additional information

No additional information is available for this paper.

## Declaration of interest's statement

The authors declare no competing interests.

## Appendix A Supplementary data

Appendix_spl_1-16_spl_ Appendix_spl_1-16_spl_

List of Supplementary Materials List of Supplementary Materials
