# Supplementary material for: An angiogenesis-related three-long non-coding ribonucleic acid signature predicts the immune landscape and prognosis in hepatocellular carcinoma
Source: Heliyon. 2023 Feb 23;9(3):e13989. doi: 10.1016/j.heliyon.2023.e13989 (PMC9982620; doi:10.1016/j.heliyon.2023.e13989)
Supplement: List of Supplementary Materials [file mmc2.docx]

# Supplementary Materials

1. Table S1. 36 angiogenesis-associated genes

2. Appendix 1. Co-expression data of angiogenesis-associated genes and related lncRNAs

3. Appendix 2. Differential expression data of angiogenesis-associated lncRNA in HCC tumor and normal samples

4. Appendix 3. Univariate Cox regression analysis data

5. Appendix 4. Risk scores for all samples

6. Appendix 5. Univariate Cox and multi-Cox regression independent prognostic analysis data for the model

7. Appendix 6. Risk data of nomograms

8. Appendix 7. Clinical parameters of HCC patients

9. Appendix 8. Data on the correlation between immune cells and risk scores

10. Appendix 9. ssGSEA data

11. Appendix 10. Cluster analysis data

12. Appendix 11. ESTIMATE Score analysis data

13. Appendix 12. Differential analysis data of immune cells in different clusters

14. Appendix 13. Clinicopathological parameter data for the CHCC cohort

15. Appendix 14. Expression, survival, and corresponding risk score data for signature lncRNAs in the CHCC cohort.

16. Appendix 15. Univariate Cox and multi-Cox regression data for the model in the CHCC cohort.

17. Appendix 16. The expression dataset of lncRNAs in this signature.
